# Defensins and Viral Infection: Dispelling Common Misconceptions

**DOI:** 10.1371/journal.ppat.1004186

**Published:** 2014-07-17

**Authors:** Mayim E. Wiens, Sarah S. Wilson, Carissa M. Lucero, Jason G. Smith

**Affiliations:** Department of Microbiology, University of Washington, Seattle, Washington, United States of America; University of Florida, United States of America

## Introduction

**Figure 1:**
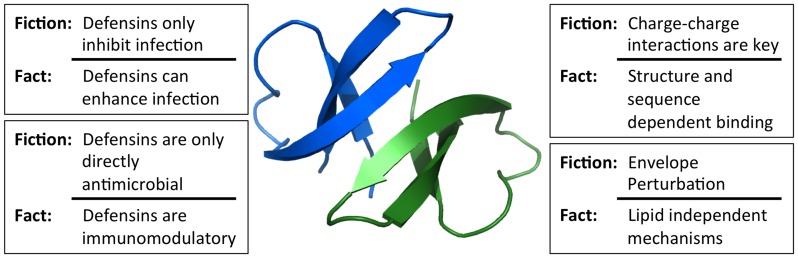
Common misconceptions of defensin antiviral activity. Studies of viral interactions with defensins have dispelled many misconceptions about the key properties of defensins that dictate their activity. The in vivo relevance of these mechanisms for viral pathogenesis has yet to be firmly established. Image of the HD5 dimer was generated using Pymol (PDB: 1ZMP).

Human α- and β-defensins are cationic, amphipathic effector peptides of the innate immune system with broad antimicrobial activity [1]. α-defensins are produced by neutrophils (human neutrophil peptides [HNP] 1–4), as well as by epithelial cells in the gut and genitourinary tract (human defensins [HD] 5 and 6). β-defensins (human β-defensins [HBD] 1–4) are constitutively expressed by epithelial cells of skin and mucosal surfaces. Originally discovered due to their antibacterial activity, defensins are also active against both enveloped and non-enveloped viruses. Mechanisms important for bacterial inhibition have been historically assumed to be responsible for defensin antiviral activity; however, this assumption has not held up to experimentation. Our purpose is to address some persistent myths regarding the activity of human defensins against viruses, as well as to identify areas requiring further investigation (Figure 1).

## Misconception: Antiviral Activity Is Only Due to Defensin Positive Charge

The initial antibacterial mechanism was proposed to be largely dependent on simple charge–charge attraction to the bacterial membrane [2]. Accordingly, defensins are most potently antibacterial in hypotonic media [1], [3]. Antiviral activity, in contrast, is generally preserved at physiological salt concentrations in normal cell culture media, arguing against a dominant charge–charge component to their interaction with viruses [4]–[6]. There is additional strong evidence that charge alone does not dictate antiviral activity: linearized α-defensins that lack a disulfide-stabilized 3-D structure are nonfunctional against all viruses tested [6]. Additionally, obligate monomer forms of α-defensins are highly attenuated for binding to and/or inactivation of viral pathogens. [7], [8]. If antiviral activity were simply due to charge–charge interactions, these defensin mutants would still be active, as their charge is conserved. A second line of investigation using direct mutational analysis showed that arginine to lysine substitutions at specific residues attenuate the antiviral activity of HD5 [7]. These results, coupled with the marked preference for arginines over lysines in α-defensins, imply that other aspects of arginine residues are more important than simple charge [6], [9]. Furthermore, β-defensins are, on average, more charged than α-defensins yet largely exhibit less antiviral activity, especially against non-enveloped viruses. Thus, while charge is important for defensin function, it is not the only determining factor in antiviral activity.

## Misconception: Defensins Only Work by Damaging the Viral Envelope

Lipid perturbation is a second key component of the canonical defensin antibacterial mechanism that was thought to extend to their antiviral activity. Indeed, an early study found that enveloped viruses were sensitive to neutrophil α-defensins, while the non-enveloped viruses included in this study were not [10]. Despite the pervasiveness of the notion that defensins perturb the lipid bilayer of enveloped viruses, there is scant direct evidence. In fact, the only clear data that supports this idea is the inhibition of respiratory syncytial virus (RSV) by HBD2 [11]. An inability of defensins to directly perturb viral lipid envelopes is consistent with the observation that cholesterol and other neutral lipids, which are commonly found in viral envelopes but not in bacterial membranes, attenuate the membrane lytic activity of defensins and other antimicrobial peptides [3], [12]. Thus, viral inhibition by defensins can occur by multiple mechanisms that are distinct from lipid perturbation, including effects on target cells rather than the virus particle [13]. Importantly, despite the initial study to the contrary, α-defensins have been shown to block infection by multiple non-enveloped viruses, which by definition lack a lipid target. For these viruses, mechanisms include extracellular aggregation, inhibition of viral uncoating, and blocking the viral genome from reaching the nucleus [6]. Overall, the inhibition of non-enveloped viruses and the myriad ways that defensins have been shown to inhibit viral infection do not support a role for lipid perturbation as the defining mechanism.

## Misconception: Defensins Exclusively Inhibit Viral Infection

Although the majority of studies have focused on the antiviral activity of defensins, in some cases, α-defensins actually enhance human immunodeficiency virus (HIV) and human adenovirus (HAdV) infection [14], [15]. For both viruses, enhancement is not observed with linearized defensins, indicating that structure-dependent interactions are required. Treatment of HIV with HD5 or HD6 substantially increases infection, which for some strains can reach >100-fold [15]. This enhancement is sufficient to overcome the effects of entry and fusion inhibitors and acts primarily by increasing viral attachment to target cells [16]. Naturally produced HD5 from *Neisseria gonorrhoeae*–infected cells also enhances HIV infection, suggesting that it is likely to occur under physiological conditions in vivo [15]. We have observed a similar, albeit much more modest, HNP1- and HD5-dependent increase in infection by certain serotypes of HAdV, which is also correlated with increased receptor-dependent and -independent attachment to cells [7], [14]. Whereas HIV is sensitive to HNPs but enhanced by HD5 and HD6, HAdV serotypes appear to be more uniformly resistant or sensitive to α-defensins in general. Given that infection by two disparate viral families is enhanced by defensins, it would not be surprising if this were true for other viruses. It also remains to be seen if enhancement occurs in vivo and whether enhancement or inhibition predominates.

## Misconception: Defensin In Vitro Activity Predicts Their Role In Vivo

In addition to direct effects on viral infection, defensins also target cells and are immunomodulatory [6], [17], [18]. Thus, even defensins that are not directly antiviral could influence the activation and function of immune cells recruited to a site of viral infection, thereby impacting viral pathogenesis in vivo. These mechanisms likely explain the immunopathology of influenza virus infection in a mouse lacking β-defensin 1 [19]. They also function when defensins are used as adjuvants by altering the numbers and specific subsets of immune cells recruited in response to model antigens [20]. Whether this adjuvant effect occurs in the context of a pathogenic infection has not been shown. Moreover, the cellular receptors for many defensins are unknown, making the mechanism by which chemotaxis occurs unclear. Separate from these immunomodulatory activities, work in animal models with altered defensin expression levels has shown that the defensin repertoire impacts the composition of the commensal microbiota [21]. A logical extension of these studies is that the commensal population shaped by defensin expression could influence susceptibility to viral infection. Although these are all mechanisms by which defensins could potentially influence viral infection, there is minimal data as to their actual effect on viral pathogenesis in vivo.

## Conclusions

Cell culture studies have provided the vast majority of the evidence demonstrating a direct antiviral effect of defensins under assay conditions that do not result in defensin-mediated cytotoxicity [22]. Indirect evidence for the importance of defensins in vivo comes from a limited number of association studies of defensin levels with viral disease states or progression [6], [13]. Although mouse models with altered or transgenic defensin expression exist, a clean genetic knockout has not been generated due to the complexity and the extent of the mouse defensin locus [23]. In addition, there is no animal model that completely recapitulates the human defensin repertoire. Mice, in particular, lack myeloid α-defensins. Due to the paucity of data, the most pressing issue in the field is to firmly establish in vivo relevance for the growing body of in vitro data characterizing the antiviral properties of these interesting and multifunctional peptides.
